# The technological side of the microbiome

**DOI:** 10.1038/npjbiofilms.2015.1

**Published:** 2015-03-25

**Authors:** Willy Verstraete

**Affiliations:** 1 Laboratory of Microbial Ecology and Technology (LabMET), Ghent University, Gent, Belgium

## The homecoming of the technologist

**Figure 1:**
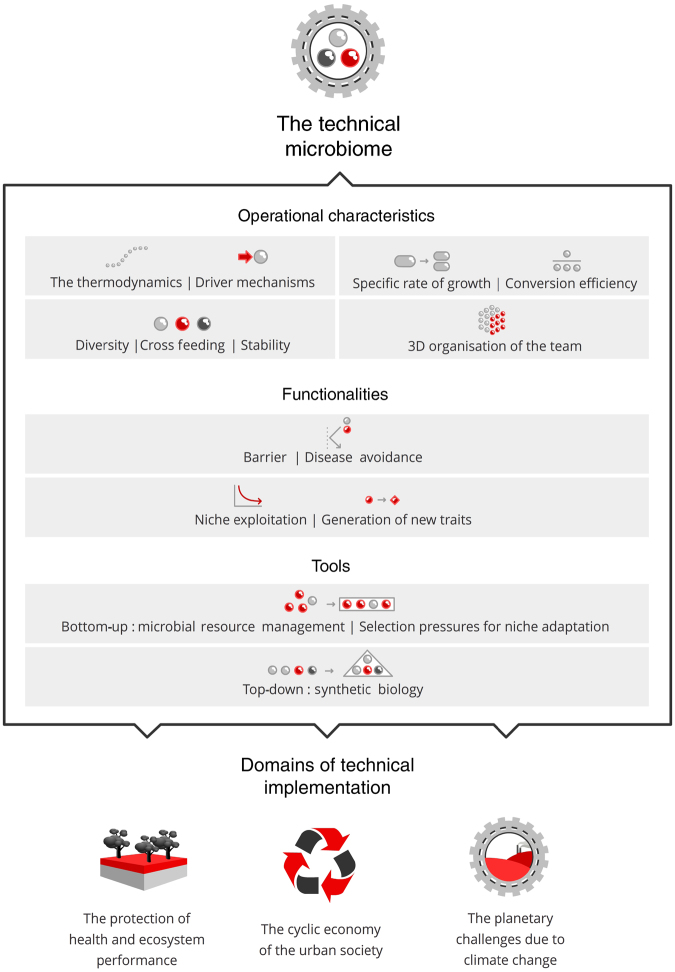
Conceptual scheme of the technicality of microbiomes in the industrial society.

Microbial ecologists have struggled for a long time with the concept of how to represent in a single comprehensive term the fact that microorganisms apparently can grow and work together. Most often one has referred to ‘mixed cultures’ (respectively ‘microbial associations or communities’). In that respect, the word ‘biofilm’ was indicative of living and working together in a structured way. Yet the term ‘microbiome’, as coined for the first time in 2005, was even more striking.^1^ Indeed, it provides a connation that does not relate to carrier or surface materials, and thus can be applicable for bio-systems operational in full-scale technical installations such as drinking water supply installations, used-water treatment systems, air scrubbers, composting plants, various types of anaerobic digester systems, bio-electrochemical configurations and soil biotreatment installations. In all these technical systems, normally one has organised communities of microbes at work and they are present in the form of 3-dimensional coagulates, flocs, sludges, granules and deposits. Also in various food-treatment facilities and zootechnical and medical devices open to microbial invasion, microbiomes are the central active principle. They bring forward changes in chemical or physical composition. In addition, they are often highly desired because they exclude unwanted species and thus have a barrier function. For these technical systems, the concept of ‘microbiome’ as the bio-catalytic actuator has been a breakthrough, as it reflects both the microbes and the collective genomes that are interacting. Clearly, the technologist dealing with the design, optimisation, operation and control of technical microbial systems has at last a term that reflects the very nature of his/her attention, i.e., the assemblage of microorganisms operating as a complex self-organising system having a level of species stability and driving particular conservation and conversion processes under open and variable conditions. The overall line of consideration with respect to the technical aspects of the microbiome in the context of the current societal challenges is depicted in Figure 1.

## The microbiome as an operational concept

The technologist needs transparent operational concepts to design. Particularly useful are parameters such as species composition, minimum cell residence time and specific loading rate. The first is often cited in the context of performance and stability of the community at work.^2^ The second reflects the rate at which the slowest growing species in the microbiome is capable to reproduce. In this way, biofilms are special since they allow for very slow organisms to decouple solid from hydraulic residence time in the system. The last deals with the fact that per unit of microbial biomass (usually expressed in dry weight) a certain amount of substrate can be converted per unit time with good efficacy. Besides such parameters, a clear cut description of the boundary conditions (standard thermodynamics in general and pH, temperature, redox potential and salinity levels in particular) is essential to properly frame the technology.^3^ All these aspects can at present be defined quite coherently to the extent that indeed industrial investments for such technical applications entirely based on microbiomes are occurring worldwide, and in fact most often function with good results notwithstanding the rather limited insight there is about their true nature and functioning.

Indeed, the technologist dealing with ‘microbiology open to the outside world’ struggles heavily with the question on how to come to grips with the core of the biology that drives the system. What is the active driver of this mixed culture of microorganisms? And more specifically, is it really a so-called team of microorganisms working together or is it a haphazard assemblage of microbes happening to be there but with little or no relation to other similar sites and situations? Indeed, there are at present no established hands-on criteria to delineate if one has a microbiome at work. One can verify by simple community fingerprinting techniques (DGGE, T-RFLP or analogous analyses) whether there is a Pareto type structuring in which some 20% of the bacteria are responsible for about 80% of the energy flux.^4^ Another approach is to screen for cross feeding patterns in which distinct groups of species provide the metabolites for subsequent groups on which they concomitantly depend for the removal of their products. A typical example is the food chain in a well-functioning methane-producing consortium. A third and more tricky element is to examine the dynamics of diversity within the microbiome. The logical hypothesis is that microbiomes succeed to maximise the ‘exergy’ (useful energy) of the overall process and hence tend to generate maximum biomass yield based on complex rather than on simple metabolic patterns, thus allowing for a multitude of species to participate in the process, i.e., the more the merrier.^2^

This notion of biodiversity for the technologist has many intriguing aspects. Indeed, the more divers a microbiome, the more stable it generally is considered to be in its functioning.^5,6^ Yet no pragmatic reference values useful to the technologist are available.

In relation to the barrier function, it has now been documented at the level of higher animals (i.e., health of amphibians in relation to flatworms in small lakes) that indeed biodiversity is inversely related to disease.^7^ There is an urgent need to further examine to what extent the level of protection against invaders of microbial communities can be increased (respectively benchmarked).^8^

In practice, the technologist is particularly keen to achieve maximum performance. At this point, the question arrives if indeed the microbiome has an advantage over constructed mixed cultures to the extent that it inherently strives for total exploitation of all niches. Recent work has demonstrated that, in activated sludges, ‘generalists’ such as for instance Microthrix species have too large a genome to allow mutations and thus must keep tight control, even under variable operational circumstances.^9,10^ It is postulated that consequently the ‘specialists’ can have higher levels of within-population variation for diversification and fine-scale niche participation.^11^ In this theoretical setting, the practitioner should strive for a good balance between the two types of organisms within the microbiome, but the tools to do so are yet neither defined nor calibrated. Furthermore, it has recently been shown that heritable genotypes resulting from distinct genotypes can be generated in communities of higher plants due to selection pressures.^12^ Theoretical models suggest that, for microbial populations, the ongoing variations maybe largely due to neutral evolutionary processes (mutation, recombination and genetic drift), but some sequence variation can relate to niche-specific adaptation.^13^ It is of great technological value to further explore when such ‘evolution’ of new traits occurs within the operational microbiome, and how the conditions can be set to enhance such developments towards new forms of life and their respective metabolomes.

## Monitoring the manufacturing microbiomes

Technology is all about transforming a set of raw materials into added value products, thereby minimising the production of ‘entropy’ normally labelled as wastes. The bio-technologist dealing with such processes currently can obtain readily a multitude of data on the overall genomics of the microbiomes, which determine these transformational processes. Yet, these data are very much focused on lists of species, genera, clades and so on present and as well on their ratios. The criterion of different ratios has recently been adequately criticised to be of restricted value.^14^ Indeed, the genomics should detect functional differences in closely related genes from sequences alone. They must preferably deal with changes that are going to occur within the microbiome in terms of capacity and traits. In that respects, microbial proteomics should become more prominent in these studies.^15^ This type of data will be of great importance for the technologist because they can constitute a kind of early warning indicator of intrinsic changes within the microbial community. In this respect, it would also be marvelous to focus not so much on catabolic genes, which are at the basis of what the technologist observes anyway, but on anabolic genes, which relate to events that are predictors for the future within the assemblage of species in operation. Finally, manufacturing is all about supply of input materials and removal of end products, and hence the characterisation of the metabolic exchange in 3D within the microbiome is of central importance for the technologist.^16^

## The engineering of the microbiome

Two technological routes are currently under development to obtain optimal benefit from microbiomes in practice.

First, there is the so-called Microbial Resource Management approach in which (in analogy with the Human Resource Management)^17,18^ one strives by careful choice of the input of existing microbiomes and the gradual evolvement of the latter to obtain a consortium of species that attains the desired performance. Under the given conditions of micro-ecology, which particularly depends on the 3D special configuration of the partners within the team,^19^ the latter aspect should be in the forefront of further development. This bottom-up approach, also labelled as microbial community engineering,^20^ has given rise in the past decades in practice to groundbreaking achievements. Some striking examples are high-rate anaerobic digestion of municipal and other solids, granular sludge, production of novel added value compounds, effective inocula for deep soil cleanup, biological air filters resilient to highly variable ammonia inputs and so on. Yet, the strategies to come to such performing microbes thus far are rather ‘archaic’. Platform strategies are lacking or ambiguous. For instance, it is not clear whether one should start from broad-based microbiomes mainly containing generalists or from those that contain mainly specialists. There are some indications that the former is advisable since the generalists are essential for the so-called biodiversity ecosystem functioning, particularly if one has only limited species redundancy,^21^ which is normally the case under such techno-industrial conditions. Also highly controversial is the question whether such up-starting microbiomes need to be regularly cross-inoculated with new and different ones (i.e., containing challenging species), which would be in line with the Hubbell^22^ theorem of neutral ecology. Finally, there is also the major practical question whether such operational microbiomes need to be challenged at regular intervals either by abiotic or biotic changes of conditions, to keep them fit for purpose.^8^

The second route is generally referred to as synthetic biology.^23^ By examination of the potential metabolomics and their regulation, one can select well-known strains as operational units and bring them to co-habitation as for instance often practiced in food technology.^24^ It stems to reason that by use of genome editing with the proper driving tools^25^ and putative robotic DNA alterations and optimisations,^26^ one will come the top down design of microbiomes of which the overall functionality is optimal, possible capable to withstand competition from the natural world, and effectively responding to the *in silico* predicted criteria of performance.

Both lines of development are powerful and very exciting. The first will probably be of major use in the domains of ‘low-value high-throughput’ technological conversions, while the second type particularly will become of predominance in the domain of zootechnical and medical applications of functionalised microbiomes.

## The platforms of technological progress

The combination of engineered microbiomes with clever high-tech is a domain that will expand rapidly in the near future. At the level of the rapidly evolving ‘mega urban society’, the latter are essential in achieving various elements of the much desired cyclic economy. Indeed, one will need to develop large-scale technologies to recycle rather than dissipate as it is now the case for the various commodities we use, even to the extent that we will need to directly recycle human waste and used waters into feed and food.^27^

One will also have to develop engineered microbiomes that effectively provide health and functional security at the interfaces of higher-lower organisms under variable conditions rather than to rely, as is at present the case, on a set of chemical/physical approaches such as total or partial disinfection, consumption of antibiotics and implementation of axenicity.^28^

Finally, in the domain of the planetary challenges and the society at large, a vast amount of technical possibilities in which well-operated microbiomes can become instrumental to improve the quality of life are waiting for exploration. The abatement of methane emissions by animals and wastes, the emission of nitrous oxide by the nitrogen-converting microbiomes, new forms of capturing CO_2_ into microbial single-cell food and so on are only a few examples to indicate the vast potentials.

Microbiomes and technology have plenty of potentials, provided the basic concepts and principles are better understood and translated into effective operation and control strategies. Happy are those who now start to explore this domain.

## References

[bib1] Backhed F, Ley RE, Sonnenburg JL, Peterson DA, Gordon JI. Host-bacterial mutualism in the human intestine. Science 2005; 307: 1915–1920. 15790844 10.1126/science.1104816

[bib2] Ho A, De Roy K, Thas O, De Neve J, Hoefman S, Vandamme P et al. The more, the merrier: heterotroph richness stimulates methanotrophic activity. ISME J 2014; 8: 1945–1948. 24785289 10.1038/ismej.2014.74 PMC4139733

[bib3] Desmond-Le Quemener E, Bouchez T. A thermodynamic theory of microbial growth. ISME J 2014; 8: 1747–1751. 24522260 10.1038/ismej.2014.7 PMC4817618

[bib4] Marzorati M, Wittebolle L, Boon N, Daffonchio D, Verstraete W. How to get more out of molecular fingerprints: practical tools for microbial ecology. Environ Microbiol 2008; 10: 1571–1581. 18331337 10.1111/j.1462-2920.2008.01572.x

[bib5] Wittebolle L, Marzorati M, Clement L, Balloi A, Daffonchio D, Heylen K et al. Initial community evenness favours functionality under selective stress. Nature 2009; 458: 623–626. 19270679 10.1038/nature07840

[bib6] Awasthi A, Singh M, Soni SK, Singh R, Kalra A. Biodiversity acts as insurance of productivity of bacterial communities under abiotic perturbations. ISME J 2014; 8: 2445–2452. 24926862 10.1038/ismej.2014.91 PMC4260711

[bib7] Richgels KLD, Hoverman JT, Johnson PTJ. Evaluating the role of regional and local processes in structuring a larval trematode metacommunity of Helisoma trivolvis. Ecography 2013; 36: 854–863.

[bib8] De Roy K, Marzorati M, Negroni A, Thas O, Balloi A, Fava F et al. Environmental conditions and community evenness determine the outcome of biological invasion. Nat Commun 2013; 4: 1383. 23340423 10.1038/ncomms2392

[bib9] Muller EEL, Pinel N, Laczny CC, Hoopmann MR, Narayanasamy S, Lebrun LA et al. Community-integrated omics links dominance of a microbial generalist to fine-tuned resource usage. Nat Commun 2014; 5: 5603. 25424998 10.1038/ncomms6603 PMC4263124

[bib10] Jon McIlroy S, Kristiansen R, Albertsen M, Michael Karst S, Rossetti S, Lund Nielsen J et al. Metabolic model for the filamentous “*Candidatus Microthrix parvicella*” based on genomic and metagenomic analyses. ISME J 2013; 7: 1161–1172. 23446830 10.1038/ismej.2013.6 PMC3660683

[bib11] Carbonero F, Oakley BB, Purdy KJ. Metabolic flexibility as a major predictor of spatial distribution in microbial communities. PLoS ONE 2014; 9: e85105. 24465487 10.1371/journal.pone.0085105 PMC3897421

[bib12] Zuppinger-Dingley D, Schmid B, Petermann JS, Yadav V, De Deyn GB, Flynn DFB et al. Selection for niche differentiation in plant communities increases biodiversity effects. Nature 2014; 515: 108–111. 25317555 10.1038/nature13869

[bib13] Wilmes P, Simmons SL, Denef VJ, Banfield JF. The dynamic genetic repertoire of microbial communities. FEMS Microbiol Rev 2009; 33: 109–132. 19054116 10.1111/j.1574-6976.2008.00144.x PMC2704941

[bib14] Lasken RS, McLean JS. Recent advances in genomic DNA sequencing of microbial species from single cells. Nat Rev Genet 2014; 15: 577–584. 25091868 10.1038/nrg3785 PMC4454502

[bib15] Wilmes P, Bond PL. Microbial community proteomics: elucidating the catalysts and metabolic mechanisms that drive the Earth’s biogeochemical cycles. Curr Opin Microbiol 2009; 12: 310–317. 19414280 10.1016/j.mib.2009.03.004

[bib16] Watrous JD, Phelan VV, Hsu C, Moree WJ, Duggan BM, Alexandrov T et al. Microbial metabolic exchange in 3D. ISME J 2013; 7: 770–780. 23283018 10.1038/ismej.2012.155 PMC3603389

[bib17] Verstraete W, Wittelbolle L, Heylen K, Vanparys B, De Vos P, Van De Wiele T et al. Microbial resource management: The road to go for environmental biotechnology. Engl Life Sci 2007; 7: 117–126.

[bib18] Verstraete W, Boon N, de Wiele T, Vlaeminck SE. Special Issue: Microbial resource management preface. Microb Biotechnol 2012; 5: 305–306. 22507068 10.1111/j.1751-7915.2012.00345.x PMC3821674

[bib19] Allison SD, Lu L, Kent AG, Martiny AC. Extracellular enzyme production and cheating in Pseudomonas fluorescens depend on diffusion rates. Front Microbiol 2014; 5: 169. 24782855 10.3389/fmicb.2014.00169 PMC3990056

[bib20] Moralejo-Garate H, Kleerebezem R, Mosquera-Corral A, van Loosdrecht MCM. Impact of oxygen limitation on glycerol-based biopolymer production by bacterial enrichments. Water Res 2013; 47: 1209–1217. 23260176 10.1016/j.watres.2012.11.039

[bib21] Gravel D, Bell T, Barbera C, Bouvier T, Pommier T, Venail P et al. Experimental niche evolution alters the strength of the diversity-productivity relationship. Nature 2011; 469: 89–92. 21131946 10.1038/nature09592

[bib22] Hubbell SP. Neutral theory in community ecology and the hypothesis of functional equivalence. Funct Ecol 2005; 19: 166–172.

[bib23] De Roy K, Marzorati M, Van den Abbeele P, Van de Wiele T, Boon N. Synthetic microbial ecosystems: an exciting tool to understand and apply microbial communities. Environ Microbiol 2014; 16: 1472–1481. 24274586 10.1111/1462-2920.12343

[bib24] Erkus O, De Jager VCL, Spus M, Van Alen-Boerrigter IJ, Van Rijswijck IMH, Hazelwood L et al. Multifactorial diversity sustains microbial community stability. ISME J 2013; 7: 2126–2136. 23823494 10.1038/ismej.2013.108 PMC3806261

[bib25] Sinkins SP, Gould F. Gene drive systems for insect disease vectors. Nat Rev Genet 2006; 7: 427–435. 16682981 10.1038/nrg1870

[bib26] Trent JM, Bittner MH, Zhang J, Wiltshire R, Ray M, Su Y et al. Use of microgenomic technology for analysis of alterations in DNA copy number and gene expression in malignant melanoma. Clin Exp Immunol 1997; 107: 33–40. 9020934

[bib27] Matassa S, Boon N, Verstraete W. Resource recovery from used water: The manufacturing abilities of hydrogen-oxidizing bacteria. Water Res 2015; 68: 467–478. 25462753 10.1016/j.watres.2014.10.028

[bib28] De Schryver P, Vadstein O. Ecological theory as a foundation to control pathogenic invasion in aquaculture. ISME J 2014; 8: 2360–2368. 24892581 10.1038/ismej.2014.84 PMC4260705

